# Supplementary material for: A cardiac-null mutation of Prdm16 causes hypotension in mice with cardiac hypertrophy via increased nitric oxide synthase 1
Source: PLoS One. 2022 Jul 21;17(7):e0267938. doi: 10.1371/journal.pone.0267938 (PMC9302805; doi:10.1371/journal.pone.0267938)
Supplement: S1 File — (DOCX) [file pone.0267938.s001.docx]

**Supporting Information**

**Supplemental Figures and Figure Legends**


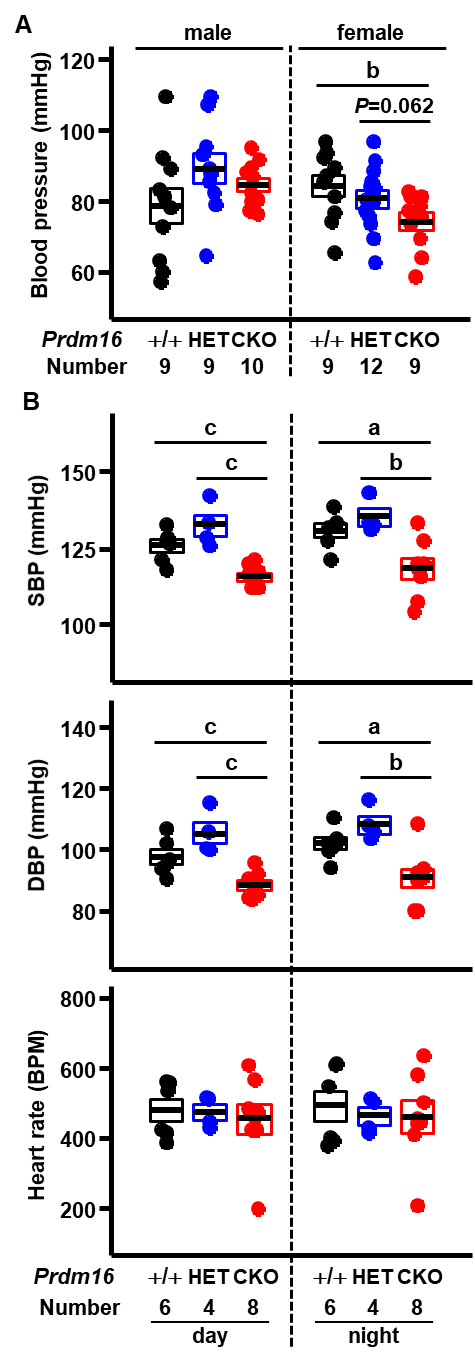


**S1 Fig.** **Decreased BP in female mice with cardiac-specific inactivation of *Prdm16*.** (A) BP was measured intra-arterially under anesthesia separately in male and female mice. (B) In female mice, SBP, DBP, and heart rate were measured via a telemetric implant for 24 hours (day and night) during conscious, unrestrained status 1 week after implantation surgery. Error bars, mean ± SEM; number (N), number of mice; statistical significance by student’s *t*-test, a (*P* < 0.05), b (*P* < 0.01), and c (*P* < 0.005).


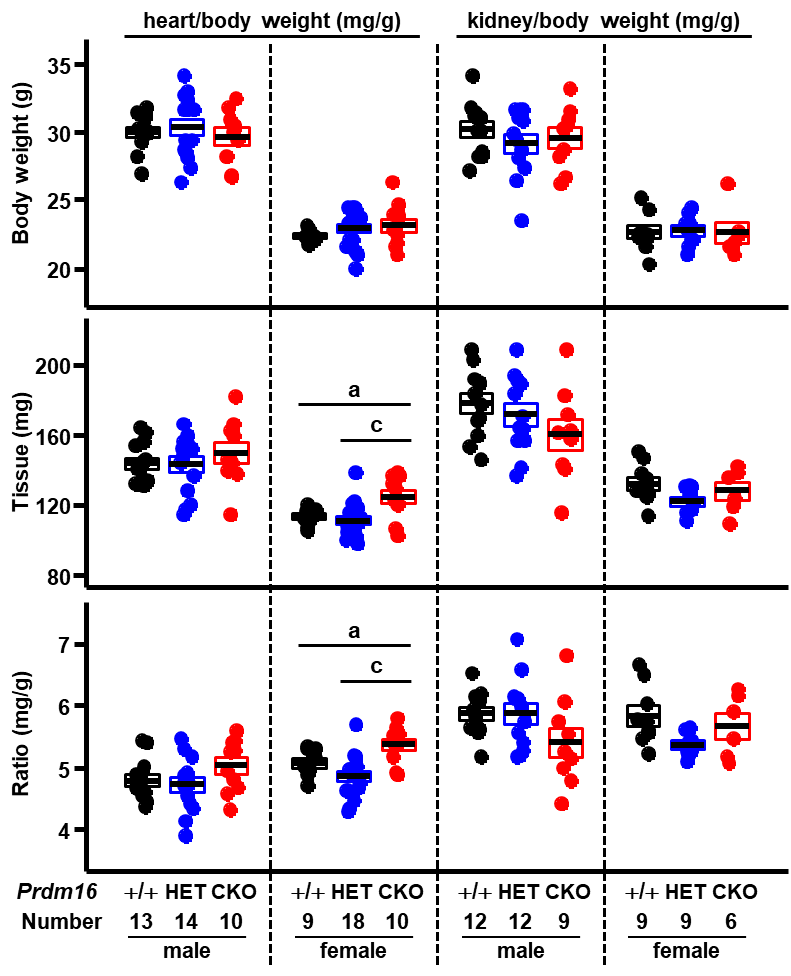


**S2 Fig.** **Changes in the ratios of heart-to-body weight and kidney-to-body weight in cardiac-specific *Prdm16* knockout mice.** Body weights, tissue (heart or kidney) weights, and ratios of heart-to-body weight or kidney-to-body weight. Error bars, mean ± SEM; number (N), number of mice; statistical significance by student’s *t*-test, a (*P* < 0.05) and c (*P* < 0.005).


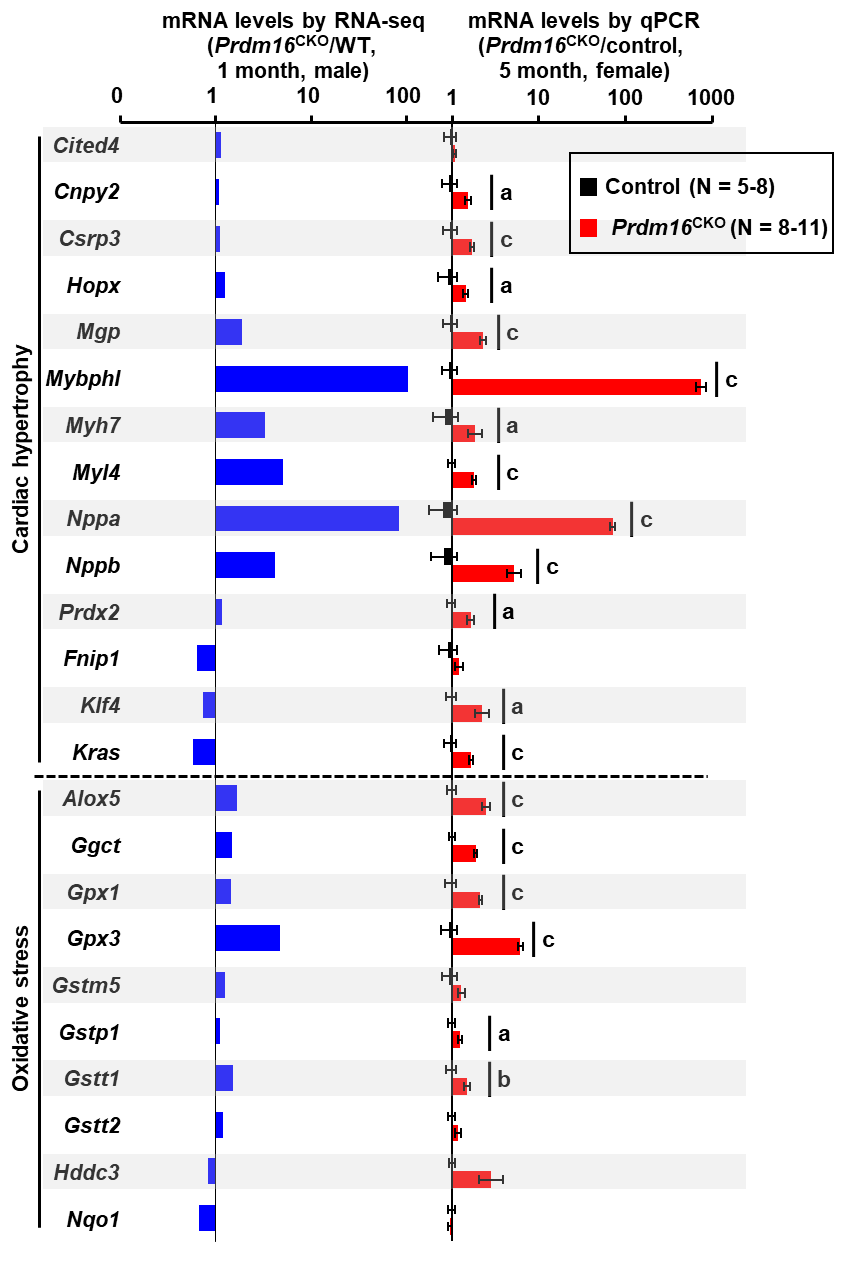


**S3 Fig. Differentially expressed transcripts in mouse hearts with a *Prdm16* deletion.** Results of RNA-seq analysis and qRT-PCR in left ventricular tissues of 1-month-old, male and 5-month-old, female mice, respectively. Cardiac hypertrophy and oxidative stress genes identified from RNA-seq analysis and validated by qRT-PCR.


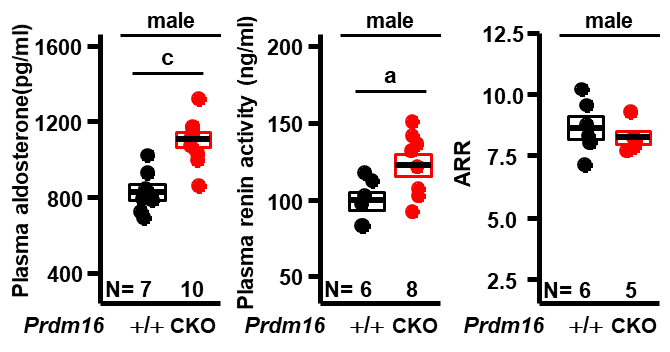


**S4 Fig.** **Changes in the aldosterone-to-renin activity ratio in male mice with cardiac-specific null mutation of *Prdm16*.** Aldosterone levels, renin activity, and aldosterone-to-renin activity ratio (ARR) in plasma. Error bars, mean ± SEM; number (N), number of mice; statistical significance by student’s *t*-test, a (*P* < 0.05), b (*P* < 0.01), and c (*P* < 0.005).


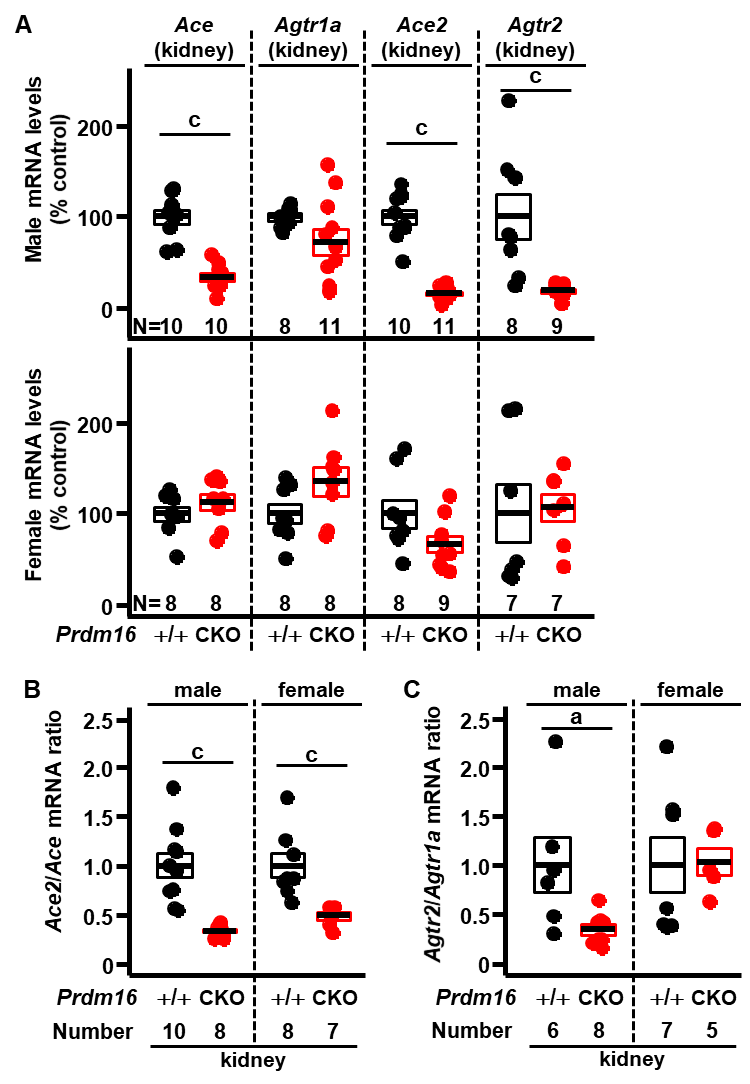


**S5 Fig.** **Changes in transcriptional expression of the renin-angiotensin-aldosterone system in mice with cardiac-specific null mutation of *Prdm16*.** (A) mRNA levels in kidney for components of the renin-angiotensin system. (B) Ratios of *Ace2*-to-*Ace* and (C) *Agtr2*-to-*Agtr1a* transcripts in kidney. Error bars, mean ± SEM; number (N), number of mice; statistical significance by student’s *t*-test, a (*P* < 0.05), b (*P* < 0.01), and c (*P* < 0.005).


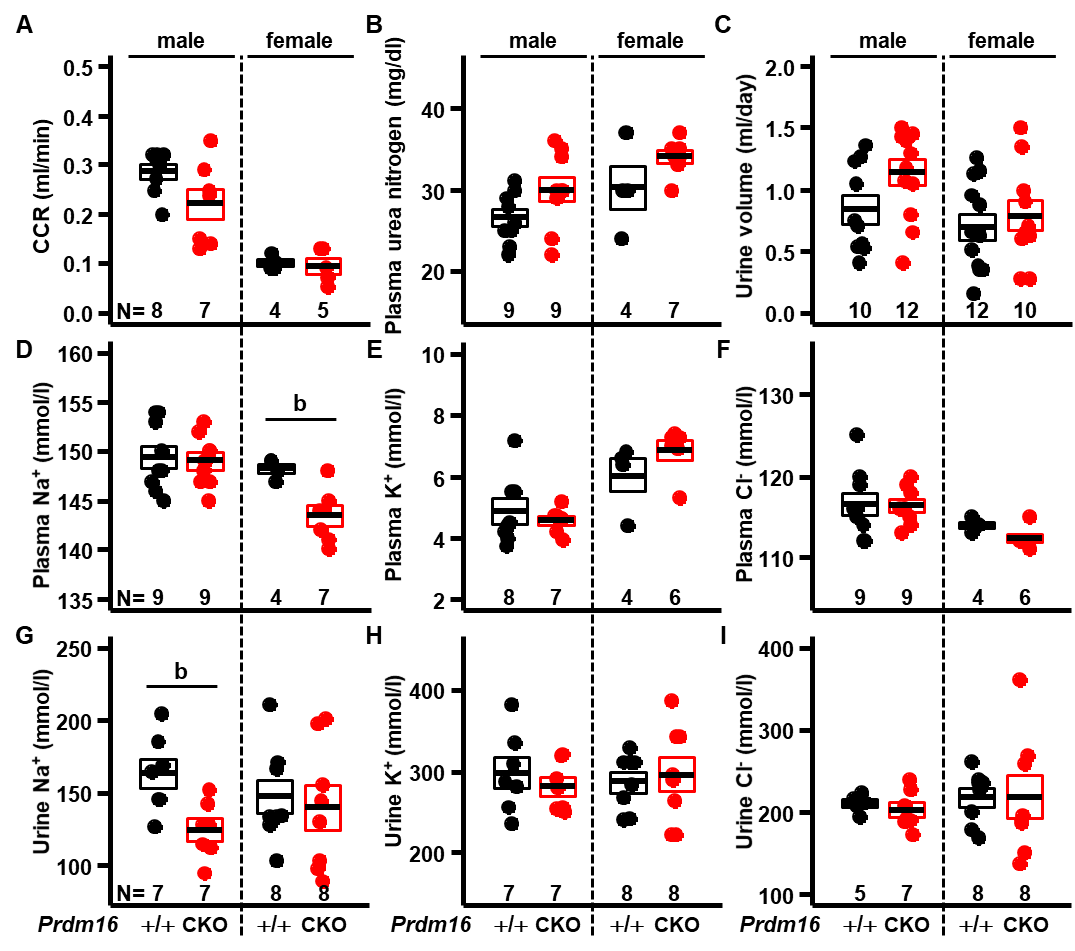


**S6 Fig.** **Changes in electrolyte balance in cardiac-specific *Prdm16* knockout mice.** (A) Creatinine clearance rate (CCR). (B) Urea nitrogen in the plasma. (C) 24-hour urine volume. (D) Plasma sodium levels. (E) Plasma potassium levels. (F) Plasma chloride levels. (G) Urinary sodium levels. (H) Urinary potassium levels. (I) Urinary chloride levels. Error bars, mean ± SEM; number (N), number of mice; statistical significance by student’s *t*-test, b (*P* < 0.01).


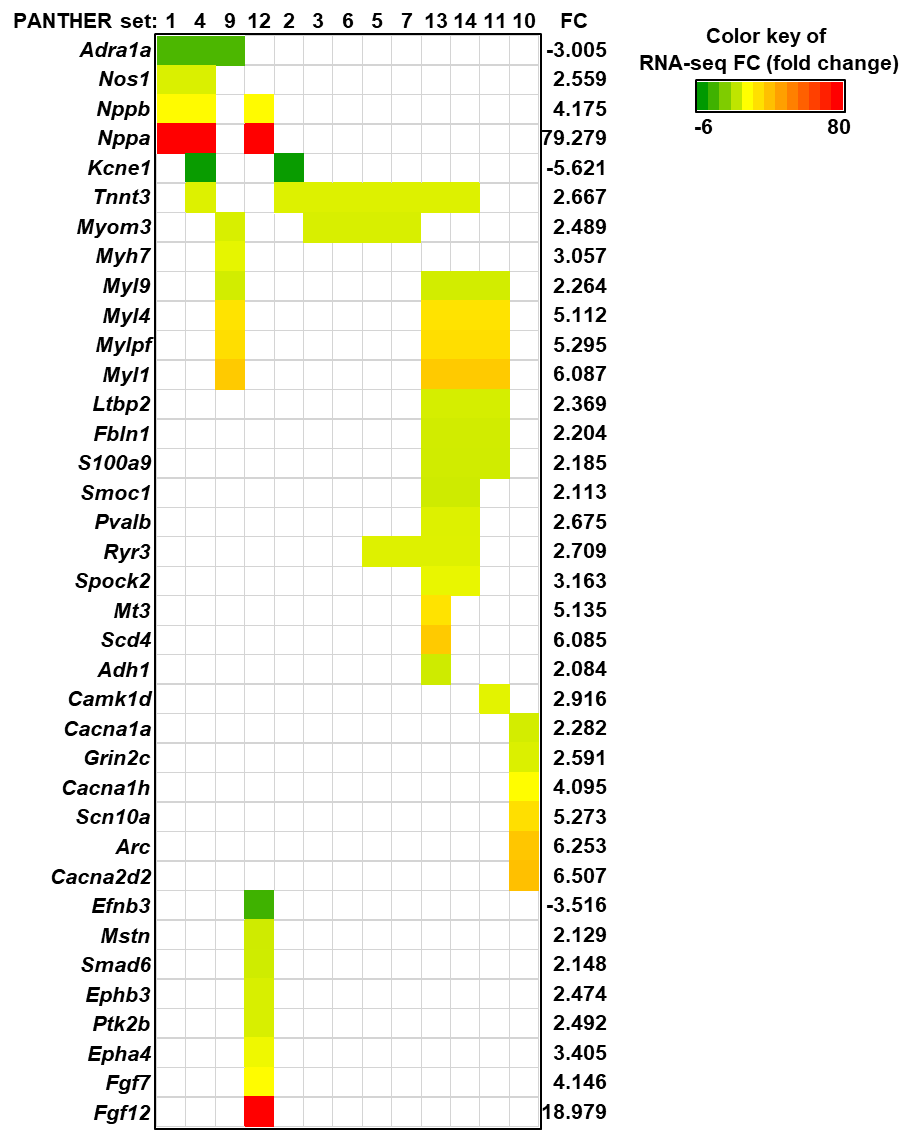


**S7 Fig.** **Visualization of functionally relevant gene sets by heat maps.** RNA-seq analysis in 1-month-old male mouse left ventricular tissue, showing 772 differentially expressed genes (DEGs)—485 upregulated and 287 downregulated—in *Prdm16*^CKO^ versus wild-type hearts. PANTHER gene ontology (GO) analysis was used for functional enrichment. Of 772 DEGs, 112 genes were categorized into 17 specific GO classes with significantly functional expression patterns (FDR P-value < 0.05, fold-enrichment > 2). The GO term “Regulation of blood pressure” ranked highest in fold-enrichment, with 4 genes (*Adra1a*, *Nos1*, *Nppa*, and *Nppb*) included. Of the functionally enriched PANTHER GO sets, genes pertaining to sets 1, 2, 3, 4, 5, 6, 7, 9, 10, 11, 12, 13, and 14 were visualized by heat maps, based on fold changes (FC) in the RNA-seq data. N, number of mice.


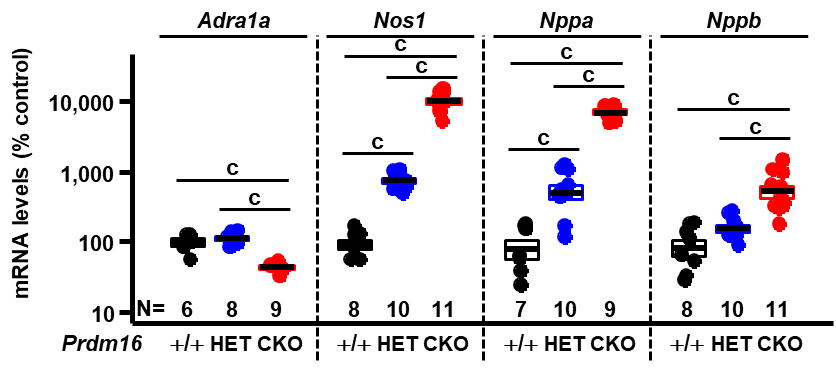


**S8 Fig.** **Transcriptional changes in 4 genes in 5-month-old, female mouse left ventricular tissue with loss of *Prdm16*.** qRT-PCR of 4 genes in 5-month-old mouse left ventricular tissue of *Prdm16*^CKO^ versus control. Error bars, mean ± SEM; number (N), number of mice; statistical significance by student’s *t*-test, c (*P* < 0.005).


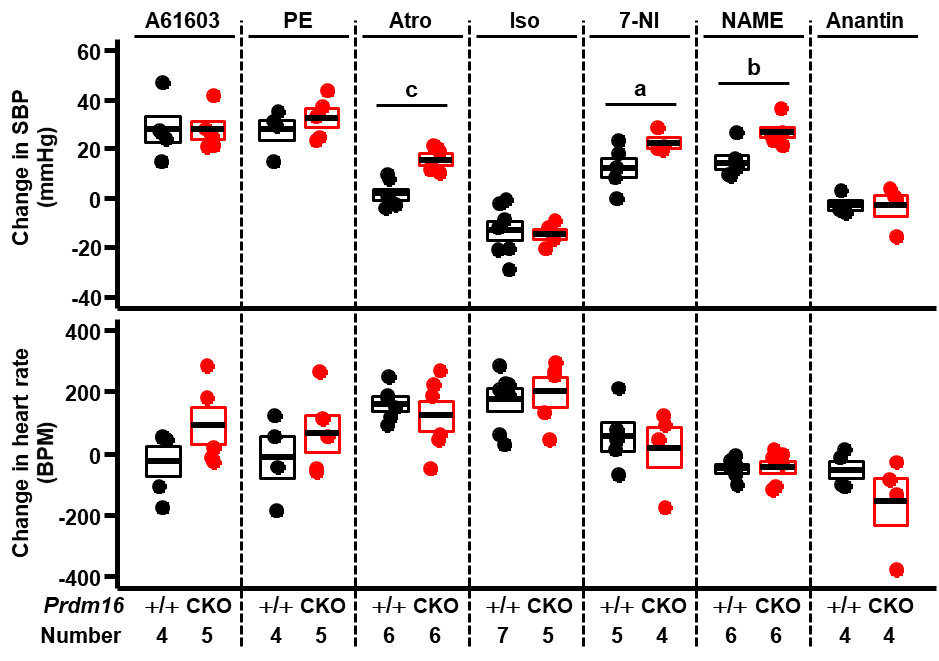


**S9 Fig.** **Changes in SBP and heart rate in mice following administration of drugs.** Changes in SBP and heart rate in female *Prdm16*^CKO^ and control mice after vehicle or pharmacological administration. Error bars, mean ± SEM; number (N), number of mice; statistical significance by student’s *t*-test, b (*P* < 0.01) and c (*P* < 0.005).


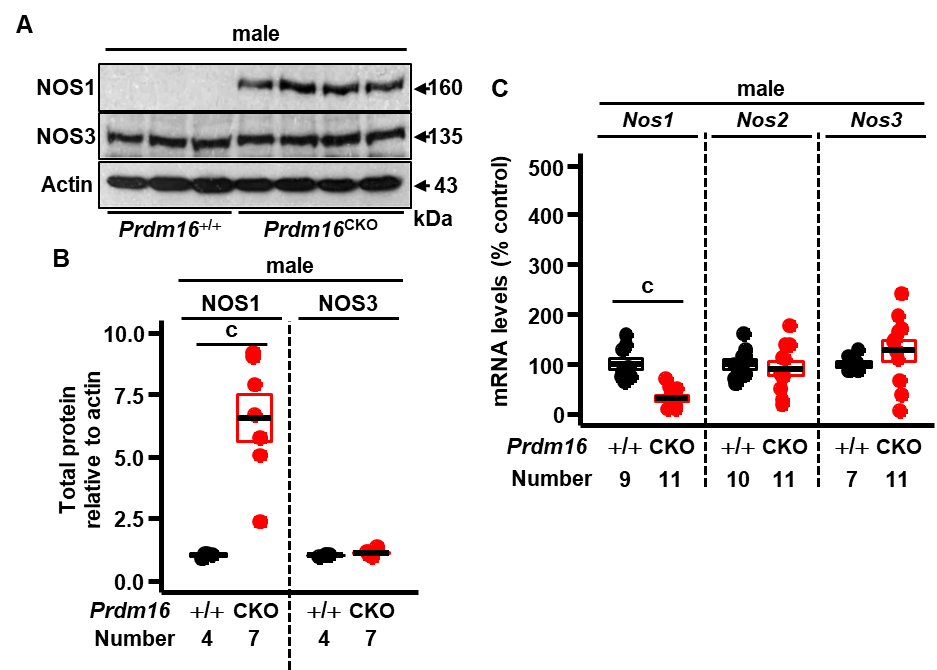


**S10 Fig. Changes in the expression of other NOS isoforms in male mice with cardiac deficiency of *Prdm16*.** (A-B) NOS1 and NOS3, as determined by western blot, in left ventricular tissue from male *Prdm16*^CKO^ and control mice. (A) Renal mRNA levels of *Nos1*, *Nos2*, and *Nos3*. Error bars, mean ± SEM; number (N), number of mice; statistical significance by student’s *t*-test, a (*P* < 0.05) and c (*P* < 0.005).

**Original images for western blots.** S1_raw_images.

**S1 Table. Primers for genotyping of the *Prdm16*^flox^ and Myh6-Cre transgenic mice**

| Mouse genotype | Forward Sequence (5'ㅡ>3') | Reverse Sequence (5'ㅡ>3') | Size (bp) |
| --- | --- | --- | --- |
| *Prdm16* flox | TATGGAGCTAGGCAGGGACA^a^ | TCCATACATCAGGGAGCAGA^b^ | ~600 |
| Wild type | TATGGAGCTAGGCAGGGACA^a^ | TCCATACATCAGGGAGCAGA^b^ | 511 |
| Myh6-Cre | ATGACAGACAGATCCCTCCTATCTCC | CTCATCACTCGTTGCATCATCGAC | ~300 |
| IPC | CAAATGTTGCTTGTCTGGTG | GTCAGTCGAGTGCACAGTTT | 200 |

IPC, internal positive control.

**S2 Table. Primers used for quantitative real-time PCR**

| Gene Symbol | Forward Primer (5'ㅡ>3') | Reverse Primer (5'ㅡ>3') | Size (bp) |
| --- | --- | --- | --- |
| *Gapdh* | GCATGGCCTTCCGTGTTC | ATGTCATCATACTTGGCAGGTTT | 85 |
| *Agt* | ATGAACTTGCCACTGGAGGG | GATGCTGTTGTCCACCCAGA | 132 |
| *Ace* | AGCCACTGACAGAATGGCTC | GCGCGAGCGGTGTTTG | 94 |
| *Ren1* | ACAGTATCCCAACAGGAGAGACAAG | GCACCCAGGACCCAGACA | 93 |
| *Agtr1a* | ACCGCTATGGAATACCGCTG | GAGACACGTGAGCAGGAACA | 105 |
| *Ace2* | AATTCCACTGAAGCTGGGCA | GGGCTCCATTCAGTGTTCCA | 200 |
| *Agtr2* | TTGGCTCCAAGGCAAGAGAG | GGCCTCCAAACCAATGGCTA | 128 |
| *Prdm16* | TGGGCTCACTACCCTACCAC | GACTTTGGCTCAGCCTTGAC | 124 |
| *Adra1a* | CGCGCAGCAGATAAACGAAG | AGTGACTCTCAACTTGGCCG | 134 |
| *Nos1* | TCGATGCCAAGGCTATGTCC | CGGACCTTGTAGCTCTTCCTC | 190 |
| *Nppa* | GCTTCCAGGCCATATTGGAG | GGGGGCATGACCTCATCTT | 126 |
| *Nppb* | ATCGGATCCGTCAGTCGTTT | CACTTCAAAGGTGGTCCCAGA | 130 |
| *Nos2* | GCTCCCTATCTTGAAGCCCC | GACACTTCGCACAAAGCAGG | 196 |
| *Nos3* | CCCAGGAGAGATCCACCTCA | CCAGGATGCAGGGCAAGTTA | 188 |
| *Alox5* | GGGCTGTAGCGAGAAGCAT | TGGTCATCTCGGGCCAATTT | 282 |
| *Cited4* | CATGGACACCGAGCTCATCG | AGCAATCGAACTCGCTCTGG | 116 |
| *Cnpy2* | GCTAGCCCTACTTTTGGGGG | GACTGGCTGCCATCTGGATT | 177 |
| *Csrp3* | TGGAGCCTGTGAAAAGACGG | CGCCCATAGCACACCTTACA | 162 |
| *Fnip1* | TGGCCCTTGCCAGAATTTGA | AGAGCTTCGATACTGATGTGTCC | 133 |
| *Ggct* | ATCTGCATGGGTGCGAAAGA | TGTCTTCCATTTCGTCGGAGA | 109 |
| *Gpx1* | CACAGTCCACCGTGTATGCC | CTTGCCATTCTCCTGGTGTCC | 230 |
| *Gpx3* | ACCAATACCTTGAACTGAATGCAC | AATTAGGCACAAAGCCCCCA | 165 |
| *Gstm5* | CTGGTTCGCCTCTGCTACAA | CTGTGCAGGTAGCTGTTCCA | 72 |
| *Gstp1* | TGTCACCCTCATCTACACCAAC | TGGGACGGTTCACATGTTCC | 299 |
| *Gstt1* | CGCGCCATTTATATCTTCGCC | ACTTGTGTGCCAGGTAGAGC | 190 |
| *Gstt2* | CCGAAAGCACAGCCATCTTG | AGTACCACGGATGTTGTCGG | 134 |
| *Hddc3* | CGGATCCTAACCCATGAGGC | TCTGACCATCCTGTAGGGGT | 299 |
| *Hopx* | CAACAAGGTCAACAAGCACCC | GGCGCTGCTTAAACCATTTCT | 101 |
| *Klf4* | AGGGAGAAGACACTGCGTCC | GATTCCTGGTGGGTTAGCGA | 260 |
| *Kras* | AGAACTGGGGAGGGCTTTCT | GCATCGTCAACACCCTGTCT | 248 |
| *Mgp* | GGCGAGCTAAAGCCCAAAAG | GTAGTCATCGCAGGCCTCTC | 80 |
| *Mybphl* | GATACACGGTGCAGAAGGCT | TTCTGGATGTGGGCAAGGTC | 190 |
| *Myh7* | CCCTCAGGTGGCTCCGAGA | AACTCTTCTTTGTCATCGGGCA | 246 |
| *Myl4* | ATCAGCCATCCTAACTGGGC | TTGGGTCAAAGGCAGAGTCC | 204 |
| *Nqo1* | CATTGCAGTGGTTTGGGGTG | TCTGGAAAGGACCGTTGTCG | 111 |
| *Prdx2* | GGCATTGCTTACAGGGGTCT | TACAGAGCGTCCCACAGGTA | 90 |

**S3 Table. Lists of antibodies used for western blotting**

| Antibody | Company | Catalogue  number | Host  species | Western blotting  dilution |
| --- | --- | --- | --- | --- |
| Actin (I-19) | Santa Cruz | sc-47778 | mouse | 1/3,000 |
| Nos1 (C7D7) | Cell Signaling | #4231 | rabbit | 1/500 |
| Nos3 | Abcam | ab66127 | rabbit | 1/1000 |
