# Supplementary material for: A cardiac-null mutation of Prdm16 causes hypotension in mice with cardiac hypertrophy via increased nitric oxide synthase 1
Source: PLoS One. 2022 Jul 21;17(7):e0267938. doi: 10.1371/journal.pone.0267938 (PMC9302805; doi:10.1371/journal.pone.0267938)
Supplement: S1 Raw images — (PDF) [file pone.0267938.s002.pdf]

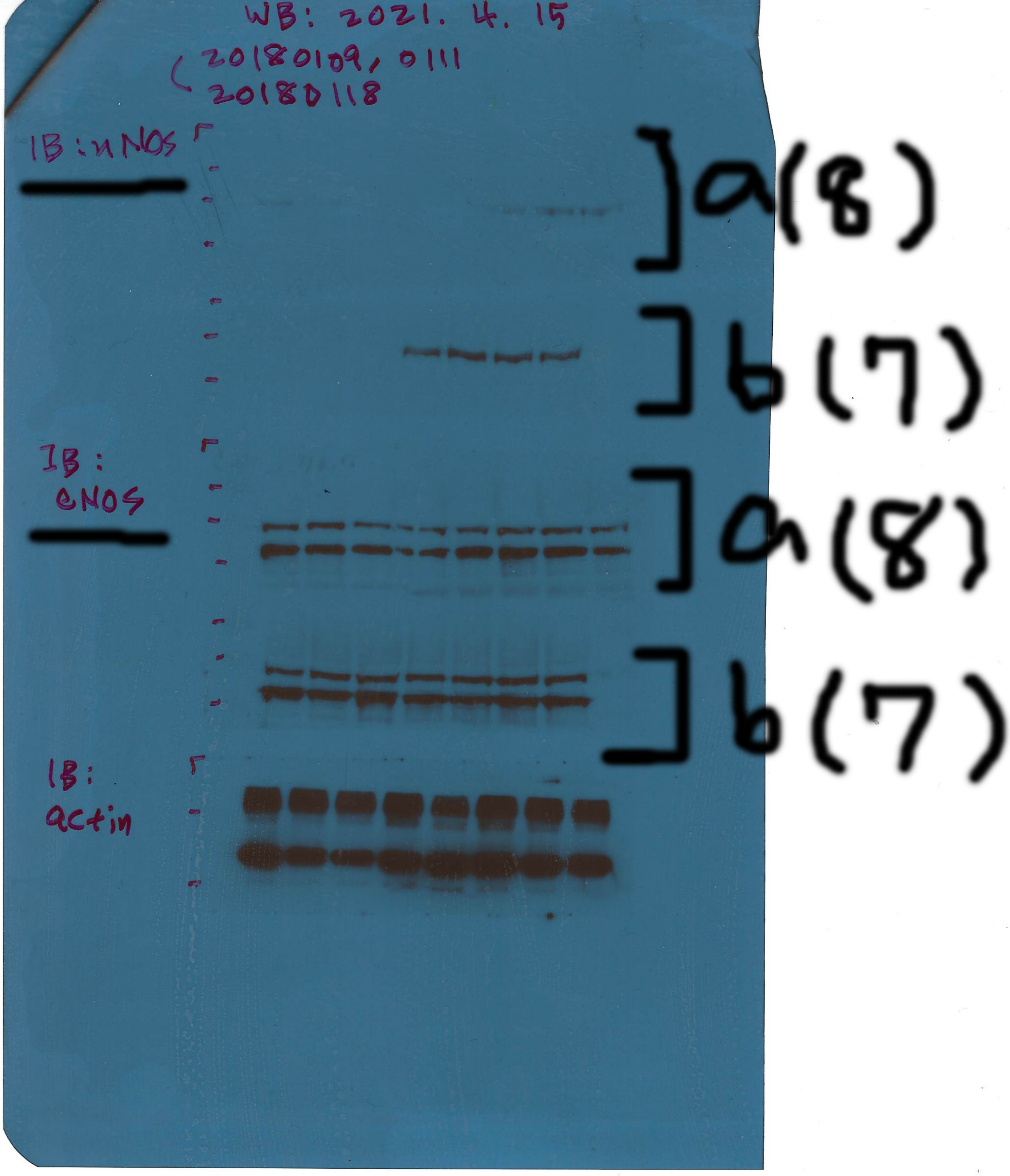
WB: 2021. 4. 15
(20180109, 0111
20180118
IB: nNOS
IB: eNOS
IB: actin
a(8)
b(7)
a(8)
b(7)

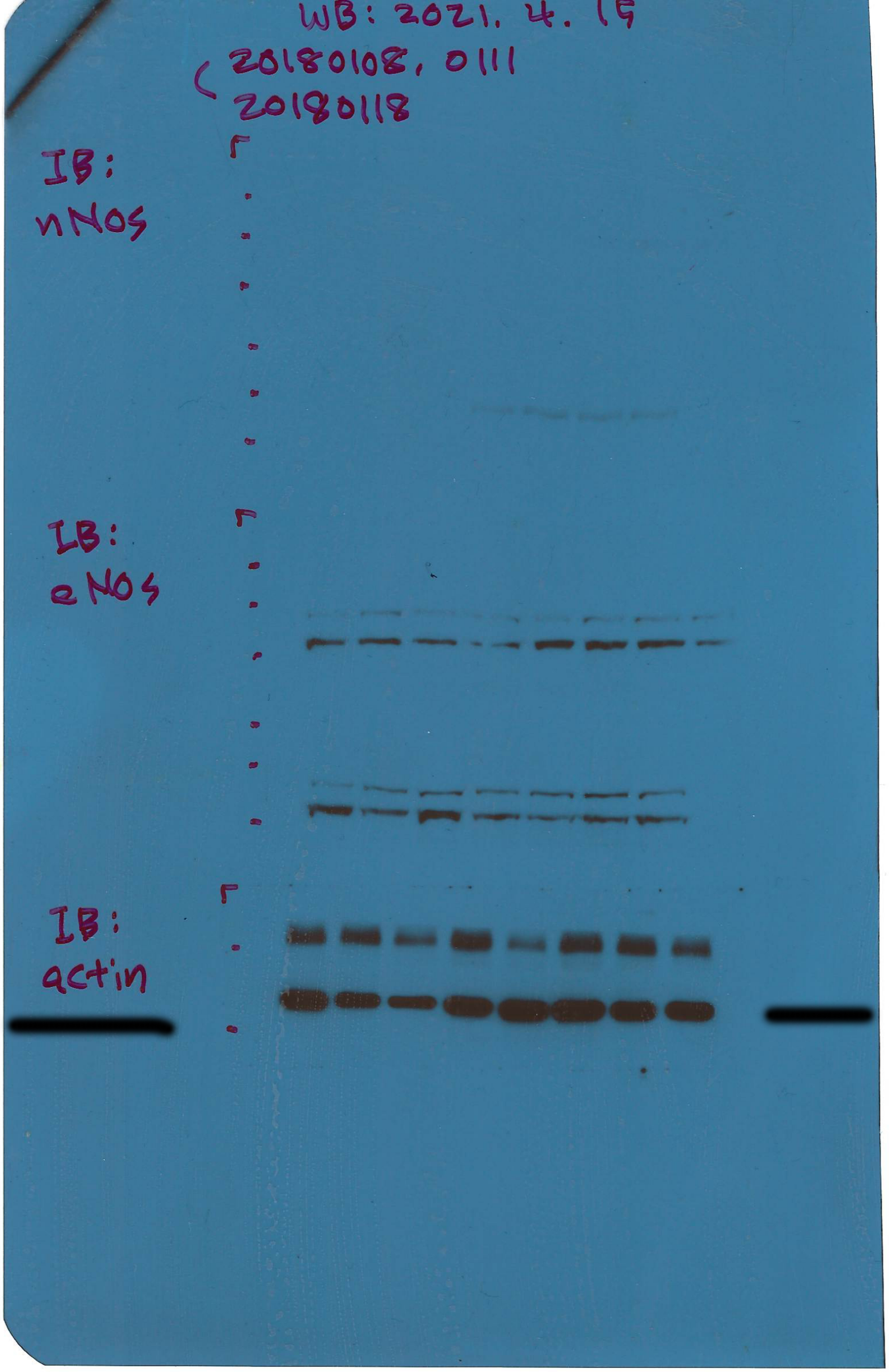

a(8)

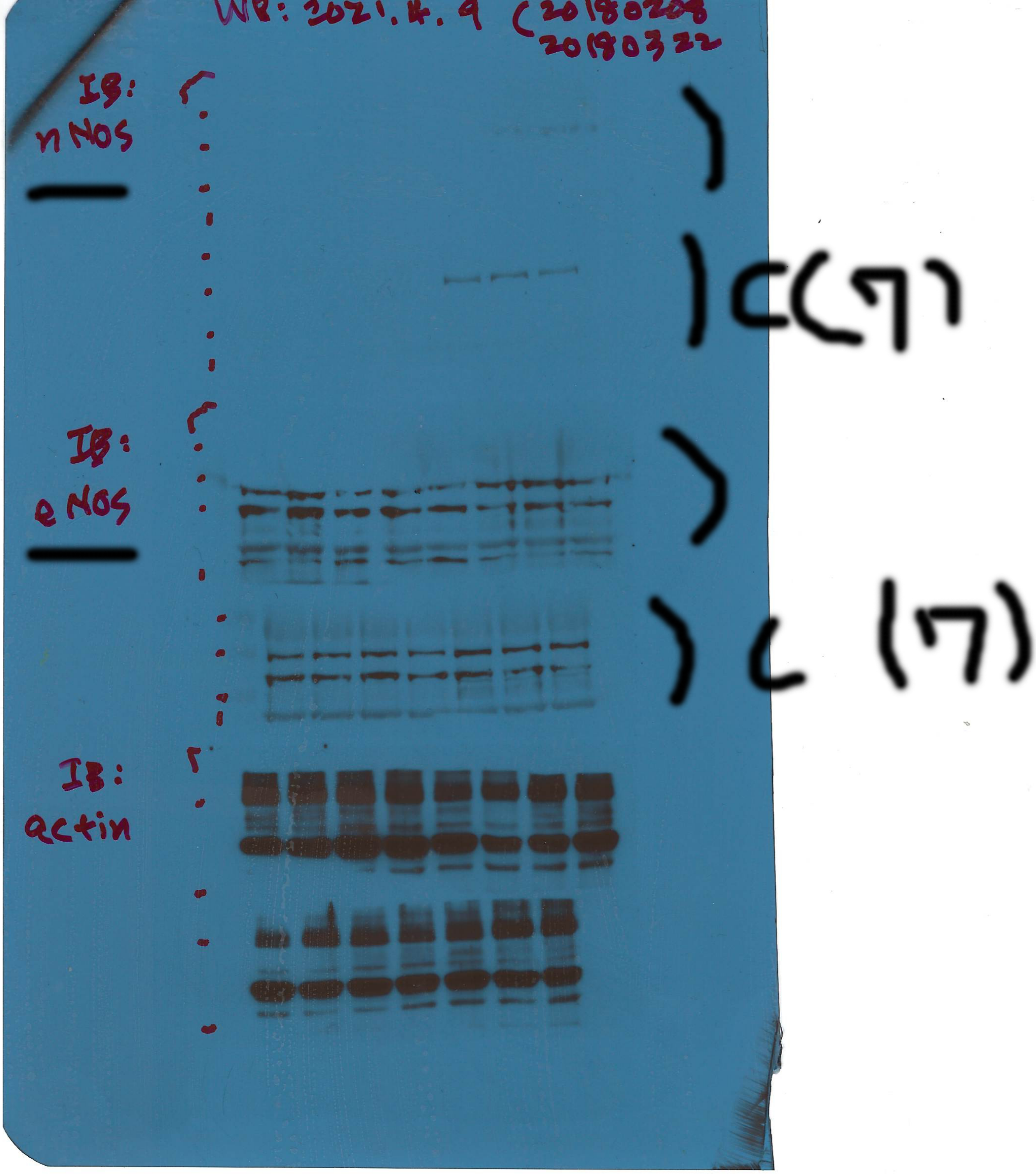

WB: 2021.4.9 (20180208
20180322
IB: nNOS
C(7)
IB: eNOS
C (7)
IB: actin

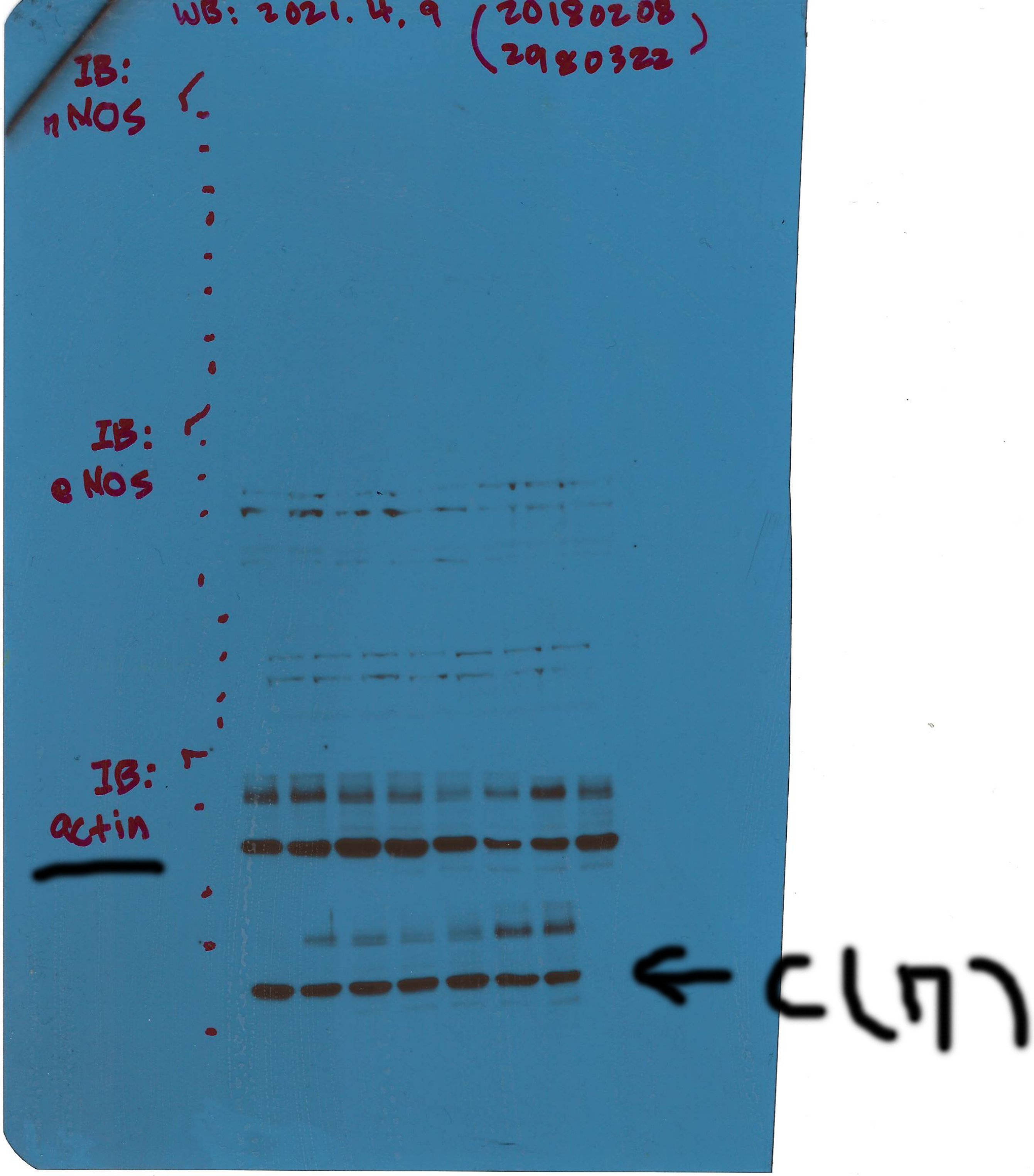
WB: 2021. 4. 9
(20180208
IB: nNOS
IB: eNOS
IB: actin
