# Supplementary material for: A cardiac-null mutation of Prdm16 causes hypotension in mice with cardiac hypertrophy via increased nitric oxide synthase 1
Source: PLoS One. 2022 Jul 21;17(7):e0267938. doi: 10.1371/journal.pone.0267938 (PMC9302805; doi:10.1371/journal.pone.0267938)
Supplement: Attachment — Submitted filename: Revision_response_to_reviewer_20220318.docx [file pone.0267938.s003.docx]

PONE-D-21-38135

A cardiac-null mutation of Prdm16 causes hypotension in mice with cardiac hypertrophy via increased nitric oxide synthase 1

PLOS ONE

Dear Dr. Kang,

Thank you for submitting your manuscript to PLOS ONE. After careful consideration, we feel that it has merit but does not fully meet PLOS ONE’s publication criteria as it currently stands. Therefore, we invite you to submit a revised version of the manuscript that addresses the points raised during the review process.

Please submit your revised manuscript by Mar 12 2022 11:59PM. If you will need more time than this to complete your revisions, please reply to this message or contact the journal office at [plosone@plos.org](mailto:plosone@plos.org). When you're ready to submit your revision, log on to https://www.editorialmanager.com/pone/ and select the 'Submissions Needing Revision' folder to locate your manuscript file.

Please include the following items when submitting your revised manuscript:

- A rebuttal letter that responds to each point raised by the academic editor and reviewer(s). You should upload this letter as a separate file labeled 'Response to Reviewers'.
- A marked-up copy of your manuscript that highlights changes made to the original version. You should upload this as a separate file labeled 'Revised Manuscript with Track Changes'.
- An unmarked version of your revised paper without tracked changes. You should upload this as a separate file labeled 'Manuscript'.

If you would like to make changes to your financial disclosure, please include your updated statement in your cover letter. Guidelines for resubmitting your figure files are available below the reviewer comments at the end of this letter.

If applicable, we recommend that you deposit your laboratory protocols in protocols.io to enhance the reproducibility of your results. Protocols.io assigns your protocol its own identifier (DOI) so that it can be cited independently in the future. For instructions see: <https://journals.plos.org/plosone/s/submission-guidelines#loc-laboratory-protocols>. Additionally, PLOS ONE offers an option for publishing peer-reviewed Lab Protocol articles, which describe protocols hosted on protocols.io. Read more information on sharing protocols at <https://plos.org/protocols?utm_medium=editorial-email&utm_source=authorletters&utm_campaign=protocols>.

We look forward to receiving your revised manuscript.

Kind regards,

Paula A. da Costa Martins, Ph.D.
Academic Editor
PLOS ONE

5. Review Comments to the Author

Please use the space provided to explain your answers to the questions above. You may also include additional comments for the author, including concerns about dual publication, research ethics, or publication ethics. (Please upload your review as an attachment if it exceeds 20,000 characters)

We greatly appreciate your (Editor’s and Reviewers’) time and efforts in reviewing our manuscript. We have tried to incorporate all of reviewer’s comments in the revised manuscript. Please find the highlighted changes in the Revised Manuscript and we provide a point-by-point response to each comment as below.

Reviewer #1: Ji-One Kang et al. described the correlation between blood pressure and heart failure in cardiac-specific Prdm16-deficient mice. Not only the authors showed that female resulted more prone to develop hypotension and cardiac hypertrophy, but also they propose that NOS1 increased expression might cause the pathogenic phenotype. The study, however, lacks the mechanistic investigation underlining this condition.

Major comments
1. On lines 266-285, the authors describe the hypertrophic phenotype in Prdm16 null mice and they report data from RNA-seq performed on 1 m.o. mouse heterozygous for Prdm16 loss. This latter model doesn’t match the one used in the study for age (1 m.o. vs 5 m.o.), sex (male vs female), and genotype (heterozygous vs homozygous). qPCR on cardiac samples of 5 m.o. female Prdm16 CKO mice should be performed to validate in the proper model the RNA-seq findings.

We agreed on your point regarding the mismatch between two mouse models. Hence, we performed qRT-PCR of those genes that are implicated in cardiac hypertrophy and oxidative stress on left ventricular tissues of 5-month-old, female *Prdm16*^CKO^ mice. Out of 24 genes, 18 genes were significantly upregulated in left ventricular tissues of 5-month-old, female *Prdm16*^CKO^ mice (S3 Fig). The results suggest that consistent with the data from young *Prdm16*^flox/flox^; Mesp1-Cre mice, cardiac hypertrophy and oxidative stress are induced in old, female *Prdm16*^CKO^ mice.

We corrected the Result section (line 274 - 291) and figure legends as follows;

In determining the molecular mechanisms that correlate with the loss of *Prdm16*, we have previously reported RNAseq data in left ventricular tissue from 1-month-old, male *Prdm16*^CKO^ and wild-type mice [1]. Consistent with the RNAseq data from young *Prdm16*^flox/flox^; Mesp1-Cre mice, we confirmed that many genes that are implicated in cardiac hypertrophy and oxidative stress were upregulated or downregulated in the cardiac tissues of 1-month-old, male *Prdm16*^CKO^ compared with control (S3 Fig). To validate the RNAseq results, we performed the qRT-PCR in the left ventricular tissue of 5-month-old, female *Prdm16*^CKO^ and controls. We found that 18 of 24 genes were significantly increased in 5-month-old, female *Prdm16*^CKO^ compared with controls, supporting the implication of cardiac hypertrophy and oxidative stress in the underlying pathological changes upon the loss of *Prdm16* (S3 Fig).

For example, the expression of natriuretic peptide A (*Nppa*), natriuretic peptide B (*Nppb*), myosin heavy chain 7 (*Myh7*), myosin light chain 4 (*Myl4*), myosin binding protein H-like (*Mybphl*), canopy FGF signaling regulator 2 (*Cnpy2*), cysteine and glycine-rich protein 3 (*Csrp3*), homeodomain-only protein X (*Hopx*), and peroxiredoxin 2 (*Prdx2*) was increased in *Prdm16*^CKO^ compared with control, suggesting that cardiac hypertrophy occurs in *Prdm16*^CKO^ (3S Fig). In addition, many antioxidant genes, such as arachidonate 5-lipoxygenase (*Alox5*), gamma-glutamyl cyclotransferase (*Ggct*), glutathione peroxidase 1 (*Gpx1*), *Gpx3*, and glutathione S-transferase (*Gst*), were upregulated in *Prdm16*^CKO^ hearts, suggesting increased oxidative stress (S3 Fig).

**S3 Fig. Differentially expressed transcripts in mouse hearts with a *Prdm16* deletion.** Results of RNA-seq analysis and qRT-PCR in left ventricular tissues of 1-month-old, male and 5-month-old, female mice, respectively. Cardiac hypertrophy and oxidative stress genes identified from RNA-seq analysis and validated by qRT-PCR.

2. In the discussion, the authors should speculate about why these findings have been observed only in female, and not male, mice

We initially thought that those BP differences between sexes should be explained in our previous version of manuscript. However, although we have accumulated multiple BP-relevant data regarding the difference between sexes, we could not draw a solid conclusion for why such BP changes have been observed only in female, but not male, mice.

However, we summarize those data regarding the BP differences between sexes and try to speculate about why such BP changes have been observed only in female, but not male, mice. Then, we tried to incorporate these supplemental data and speculations regarding sex differences in BP changes in the Result and Discussion section.

Firstly, as described in the Result section, we found that in female mice, plasma aldosterone levels and plasma renin activity did not change between *Prdm16*^CKO^ and controls, whereas the plasma aldosterone-to-renin activity ratio (APR) decreased significantly, indicating hypotensive activity (Fig 3A, B, and C). However, in male mice, plasma aldosterone levels and plasma renin activity increased significantly in *Prdm16*^CKO^ compared with controls, whereas the plasma aldosterone-to-renin activity ratio (APR) did not change between them (S4 Fig).

Secondly, we examined the indexes of kidney function in female and male *Prdm16*^CKO^ mice. Creatinine clearance rate, plasma urea nitrogen, and body weight-normalized renal weight did not differ between *Prdm16*^CKO^ and control mice, irrespective of sex (S2 and S6 Fig), indicating that kidney function was normal in *Prdm16*^CKO^ mice. Plasma potassium, plasma chloride, urinary potassium, and urinary chloride were unchanged between *Prdm16*^CKO^ and control mice (S6 Fig). Plasma sodium was lower only in female *Prdm16*^CKO^ versus control mice, but urinary sodium was lower only in male *Prdm16*^CKO^ compared with the control, although urine volume was similar in *Prdm16*^CKO^ and control animals (S6 Fig), implying that sodium reabsorption is decreased in female *Prdm16*^CKO^ mice.

Thirdly, we examined the depressor branch of RAAS pathways (ACE2/AT2R). Because sex hormones tend to favor the depressor branch of RAAS pathways in female,[2] wherein estrogen increases the depressor pathway, such as ACE2/Ang1-7/AT2R, whereas testosterone amplifies the classical ACE/AngII/AT1R pressor pathway.[3] In our study, we measured the changes in transcriptional ratios of the depressor pathway, ACE2/AT2R, versus the pressor pathway, ACE/AT1R. The relative AT2R-to-AT1R ratio decreased significantly in kidney tissue of male *Prdm16*^CKO^ mice, but did not change in female *Prdm16*^CKO^ mice (S5 Fig). These results imply that the depressor branch of RAAS pathways may play a partial role in the hypotensive activity in female *Prdm16*^CKO^ mice.

Lastly, to determine whether increased NOS expression was due to another cardiac constitutive NOS isoform, we measured NOS1 and NOS3 in left ventricular tissue by western blot.[4] NOS1 but not NOS3 rose significantly in *Prdm16*^CKO^ mouse heart tissue compared with control, suggesting that NOS1 is the major isoform (Fig 6B and C; S10 Fig A and B). In kidney, *Nos1* mRNA decreased significantly in male *Prdm16*^CKO^, and *Nos2* and *Nos3* increased in female *Prdm16*^CKO^ mice compared with control (Fig 6D; S10 Fig C). These results indicate that L-NAME-dependent restoration of hypotension is attributed primarily to the inhibition of NOS1 in heart, although the contributions by renal NOS2 and NOS3 explain in part the greater increase on treatment of *Prdm16*^CKO^ mice with L-NAME.

Besides, the AT2R exerts its depressor activity through a pathway that is mediated by NO.[5-8] In kidney cells, AT2R downregulates AT1R expression and function, dependent on NO and cGMP.[8, 9] Based on previous reports and our findings, we speculate that the induction of renal NOS2 and NOS3 as well as cardiac NOS1 contributes to the hypotensive activity in female, but not in male, *Prdm16*^CKO^ mice.

Result (line 298 - 301):

However, in male mice, plasma aldosterone levels and plasma renin activity increased significantly in *Prdm16*^CKO^ compared with controls, whereas the plasma aldosterone-to-renin activity ration (APR) did not change between them (S4 Fig).

Result (line 308 – 316):

We examined the depressor branch of RAAS pathways (ACE2/AT2R) in both male and female mice. Because sex hormones tend to favor the depressor branch of RAAS pathways in female,[2] wherein estrogen increases the depressor pathway, such as ACE2/Ang1-7/AT2R, whereas testosterone amplifies the classical ACE/AngII/AT1R pressor pathway.[3] In our study, we measured the changes in transcriptional ratios of the depressor pathway, ACE2/AT2R, versus the pressor pathway, ACE/AT1R. The relative AT2R-to-AT1R ratio decreased significantly in kidney tissue of male *Prdm16*^CKO^ mice, but did not change in female *Prdm16*^CKO^ mice (S5 Fig). This results imply that the depressor branch of RAAS pathways may play a partial role in the hypotensive activity in female *Prdm16*^CKO^ mice.

Result (line 324 – 327):

Plasma sodium was lower in female *Prdm16*^CKO^ versus control mice, but urinary sodium was lower only in male *Prdm16*^CKO^ compared with the control, although urine volume was similar between groups (Fig 4B and Ci; S6 Fig). These results imply that reduced sodium reabsorption contributes in part to the hypotensive activity in female *Prdm16*^CKO^ mice.

Result (line 376 – 377):

NOS1 but not NOS3 rose significantly in *Prdm16*^CKO^ mouse heart tissue compared with control, suggesting that NOS1 is the major isoform (Fig 6B and C; S10 Fig A and B). In kidney, *Nos2* and *Nos3* levels were higher in female, but not male, *Prdm16*^CKO^ mice versus control (Fig 6D; S7 Fig C).

Discussion (line 460 – 467):

Although the sexual differences in BP changes were not of our major concern, we tried to speculate about why such BP changes have been observed only in female, but not in male, *Prdm16*^CKO^ mice. As already described in the Result, we found that multiple results have supported a difference in BP changes between female and male *Prdm16*^CKO^ mice, including the decrease in aldosterone-to-renin ratio and sodium reabsorption, and the stable activity of the depressor branch (AT2R) of RAAS pathways only in female *Prdm16*^CKO^ mice. Based on previous reports and our findings, we can speculate that the induction of renal NOS2 and NOS3 as well as cardiac NOS1 contributes to the hypotensive activity in female, but not male, *Prdm16*^CKO^ mice.

**S4 Fig.** **Changes in the aldosterone-to-renin activity ratio in male mice with cardiac-specific null mutation of *Prdm16*.** Aldosterone levels, renin activity, and aldosterone-to-renin activity ratio (ARR) in plasma. Error bars, mean ± SEM; number (N), number of mice; statistical significance by student’s *t*-test, a (*P* < 0.05), b (*P* < 0.01), and c (*P* < 0.005).

**S5 Fig.** **Changes in transcriptional expression of the renin-angiotensin-aldosterone system in mice with cardiac-specific null mutation of *Prdm16*.** (A) mRNA levels in kidney for components of the renin-angiotensin system. (B) Ratios of *Ace2*-to-*Ace* and (C) *Agtr2*-to-*Agtr1a* transcripts in kidney. Error bars, mean ± SEM; number (N), number of mice; statistical significance by student’s *t*-test, a (*P* < 0.05), b (*P* < 0.01), and c (*P* < 0.005).

**S6 Fig.** **Changes in electrolyte balance in cardiac-specific *Prdm16* knockout mice.** (A) Creatinine clearance rate (CCR). (B) Urea nitrogen in the plasma. (C) 24-hour urine volume. (D) Plasma sodium levels. (E) Plasma potassium levels. (F) Plasma chloride levels. (G) Urinary sodium levels. (H) Urinary potassium levels. (I) Urinary chloride levels. Error bars, mean ± SEM; number (N), number of mice; statistical significance by student’s *t*-test, b (*P* < 0.01).

**S10 Fig. Changes in the expression of other NOS isoforms in male mice with cardiac deficiency of *Prdm16*.** (A-B) NOS1 and NOS3, as determined by western blot, in left ventricular tissue from male *Prdm16*^CKO^ and control mice. (A) Renal mRNA levels of *Nos1*, *Nos2*, and *Nos3*. Error bars, mean ± SEM; number (N), number of mice; statistical significance by student’s *t*-test, a (*P* < 0.05) and c (*P* < 0.005).

Minor comments
1. In figure legends, please add the explanation of f/f

We accepted your comment and the next reviewer’s comment, and then changed the figure label as control (+/+, *Prdm16*^+/+^; Myh6-Cre) and cardiac-specific knockout mice of *Prdm16* (CKO, *Prdm16*^flox/flox^; Myh6-Cre). Accordingly, we corrected the figure labels and legends as follows.

Figure legends:

**Fig 1.** **Decreased BP in female mice with cardiac-specific inactivation of *Prdm16*.** (A) BP was measured intra-arterially under anesthesia separately in 5-month-old male and female control (+/+, *Prdm16*^+/+^; Myh6-Cre) and cardiac-specific knockout mice of *Prdm16* (CKO, *Prdm16*^flox/flox^; Myh6-Cre), termed *Prdm16*^CKO^.

2. In Fig. S2 please report the unit of heart/ and kidney/body weight ratios

Yes, we added the unit of heart/ and kidney/body weight ratios as “mg/g” in the figure.

3. Fig 4Ai is a partial repetition of Fig S2

Yes, it is a partial repetition. But in the Fig S2, we wanted to show 3 genotypes and both sexes in one panel for easy comparison between genotypes and between sexes. Hence, we hope that it is acceptable.

Reviewer #2: The authors report on the axis Prdm16 and NOS1 and its role in hypotension and HF. The study is sound and the experimental setting logical and well performed There are however some points that deserve more attention:

- it is not clear whether the RNA seq data is available in any kind of repository and this available to the scientific community.

Yes, we deposited the RNA-seq data in the following site (<https://doi.org/10.6084/m9.figshare.10282136>) of which the contents include the supplementary data of our previous report.

- regarding the RNA seq data in figure 5, why not showing a heatmap with most up and downregulated genes that are relevant for this manuscript? I think this would be more explanatory and also provide additional information.

We added the heat map figure in the supplemental information and figure legends as follows.

**S7 Fig.** **Visualization of functionally relevant gene sets by heat maps.** RNA-seq analysis in 1-month-old male mouse left ventricular tissue, showing 772 differentially expressed genes (DEGs)—485 upregulated and 287 downregulated—in *Prdm16*^CKO^ versus wild-type hearts. PANTHER gene ontology (GO) analysis was used for functional enrichment. Of 772 DEGs, 112 genes were categorized into 17 specific GO classes with significantly functional expression patterns (FDR P-value < 0.05, fold-enrichment > 2). The GO term “Regulation of blood pressure” ranked highest in fold-enrichment, with 4 genes (*Adra1a*, *Nos1*, *Nppa*, and *Nppb*) included. Of the functionally enriched PANTHER GO sets, genes pertaining to sets 1, 2, 3, 4, 5, 6, 7, 9, 10, 11, 12, 13, and 14 were visualized by heat maps, based on fold changes (FC) in the RNA-seq data. N, number of mice.

- a more complete characterization of the cardiac phenotypes is necessary: histology (H&E, fibrosis, cell size), qunatification of cell size, capillary rarefaction, cross sectional stainings to see whether there is any changes in the ventricle wall, etc

In our previous report,[1] we had characterized the cardiac phenotypes by histology (H&E, fibrosis, and cell size) and quantification of cardiomyocytes. Here, we summarized the findings regarding the cardiac phenotypes.

First, *Prdm16*^CKO^ hearts appeared to be elongated longitudinally at all ages (1-, 2-, and 5-month-old) (Revision Fig 1A; Fig 2A). To estimate cardiac fibrosis, we analyzed left ventricular heart sections by trichrome staining. The accumulation of interstitial collagen was higher in all 1-, 2-, and 5-month-old heart sections of *Prdm16*^CKO^ versus wild-type mice, based on the densitometry of the collagen-positive area (Revision Fig 1B, Ci, ii, and iv). To calculate the amount of collagen directly, the amount of hydroxyproline was measured in 5-month-old heart tissue hydrolysates using a colorimetric assay kit. There was significantly more hydroxyproline in *Prdm16*^CKO^ versus wild-type heart tissue (Revision Fig 1D).

Next, to examine cellular hyperplasia, we analyzed cardiomyocyte cross-sections in trichrome-stained sections. We noted a significantly greater cardiomyocyte cross-sectional area in *Prdm16*^CKO^ compared with wild-type heart sections at all ages (Revision Fig 1Ciii, v, and 2A and B).

Next, left ventricular sections were analyzed by immunohistochemistry. *Prdm16*^CKO^ hearts showed no change in DNA-fragmented apoptosis by TUNEL assay (Revision Fig 3A and B) or in the expression of cleaved poly [ADP-ribose] polymerase (PARP) versus wild-type hearts (Revision Fig 3Ei and ii), suggesting that apoptosis does not contribute to the deteriorating phenotype of *Prdm16*-null hearts. Instead, proliferation increased in *Prdm16*-mutant hearts, as shown by immunohistochemistry using α-proliferating cell nuclear antigen (PCNA) (Revision Fig 3A and D).

Then, to detect myofibroblast expression, left ventricular sections were analyzed by immunohistochemistry using α-SMA. α-SMA was not observed in wild-type cardiomyocytes but was highly expressed in 5-month-old *Prdm16*^CKO^ hearts, based on the densitometry of the α-SMA-stained area, suggesting a myofibroblast phenotype (Revision Fig 3A and C). Collectively, the rise in *Ctgf*, *Timp1*, and *Acta2* transcripts and the induction of a myofibroblast phenotype suggests that increased collagen synthesis leads to the accumulation of collagen. To determine whether downstream signaling of TGF-β was stimulated, we examined the activation of Smad2, an intracellular mediator of TGF-β signaling, by western blot. Phosphorylated Smad2 was significantly increased in 5-month-old *Prdm16*^CKO^ compared with wild-type ventricles, as shown by quantitative densitometry, indicating stimulation of TGF-β signaling (Revision Fig 3Ei and ii).

Based on these results, we concluded that mice with cardiac null mutation of *Prdm16* presented cardiomyopathy-associated phenotypes, including fibrosis and cellular hypertrophy.

- related to the above point in figure 2 heart weight to liver weight or to tibia length should be added.

Unfortunately, we did not measure the tibia length when we collected samples. Moreover, it would take many months to get more than 3 female *Prdm16*^CKO^ mice to re-generate these data. We believe that although tibia length is the preferred index in small animal research, the body weight is very often used for normalization[10] and is not a completely wrong index unless specific conditions such as edema are noted. We can also provide another set of heart weight/body weight ratios of mice from the previous study[1] (Revision Figure 4) that do not overlap with animals of our present study, confirming the present result.

- Figure 4: give symbols to mark statistical significant instead of letters

For consistency with other figures, statistical significance was given letters such as a (*P* < 0.05), b (*P* < 0.01), and c (*P* < 0.005).

- all the mice used in the study are Myh6-Cre, there is no need to include this in all the figures. Just remove this and the figures will become more straightforward.

We accepted your comment, and then changed the figure label as control (+/+, *Prdm16*^+/+^; Myh6-Cre) and cardiac-specific knockout mice of *Prdm16* (CKO, *Prdm16*^flox/flox^; Myh6-Cre). Accordingly, we corrected the figure labels and legends as follows.

**Fig 1.** **Decreased BP in female mice with cardiac-specific inactivation of *Prdm16*.** (A) BP was measured intra-arterially under anesthesia separately in 5-month-old male and female control (+/+, *Prdm16*^+/+^; Myh6-Cre) and cardiac-specific knockout mice of *Prdm16* (CKO, *Prdm16*^flox/flox^; Myh6-Cre), termed *Prdm16*^CKO^.

**Reference**

1.	Nam JM, Lim JE, Ha TW, Oh B, Kang JO. Cardiac-specific inactivation of Prdm16 effects cardiac conduction abnormalities and cardiomyopathy-associated phenotypes. Am J Physiol Heart Circ Physiol. 2020;318(4):H764-H77. doi: 10.1152/ajpheart.00647.2019.

2.	Sandberg K, Ji H. Why can't a woman be more like a man?: Is the angiotensin type 2 receptor to blame or to thank? Hypertension. 2008;52(4):615-7. doi: 10.1161/HYPERTENSIONAHA.108.115063.

3.	Hilliard LM, Sampson AK, Brown RD, Denton KM. The "his and hers" of the renin-angiotensin system. Curr Hypertens Rep. 2013;15(1):71-9. doi: 10.1007/s11906-012-0319-y.

4.	Loyer X, Oliviero P, Damy T, Robidel E, Marotte F, Heymes C, et al. Effects of sex differences on constitutive nitric oxide synthase expression and activity in response to pressure overload in rats. Am J Physiol Heart Circ Physiol. 2007;293(5):H2650-8. doi: 10.1152/ajpheart.00883.2007.

5.	Carey RM, Jin X, Wang Z, Siragy HM. Nitric oxide: a physiological mediator of the type 2 (AT2) angiotensin receptor. Acta Physiol Scand. 2000;168(1):65-71. doi: 10.1046/j.1365-201x.2000.00660.x.

6.	Abadir PM, Carey RM, Siragy HM. Angiotensin AT2 receptors directly stimulate renal nitric oxide in bradykinin B2-receptor-null mice. Hypertension. 2003;42(4):600-4. doi: 10.1161/01.HYP.0000090323.58122.5C.

7.	Sampson AK, Moritz KM, Jones ES, Flower RL, Widdop RE, Denton KM. Enhanced angiotensin II type 2 receptor mechanisms mediate decreases in arterial pressure attributable to chronic low-dose angiotensin II in female rats. Hypertension. 2008;52(4):666-71. doi: 10.1161/HYPERTENSIONAHA.108.114058.

8.	Yang J, Chen C, Ren H, Han Y, He D, Zhou L, et al. Angiotensin II AT(2) receptor decreases AT(1) receptor expression and function via nitric oxide/cGMP/Sp1 in renal proximal tubule cells from Wistar-Kyoto rats. J Hypertens. 2012;30(6):1176-84. doi: 10.1097/HJH.0b013e3283532099.

9.	Savoia C, Ebrahimian T, He Y, Gratton JP, Schiffrin EL, Touyz RM. Angiotensin II/AT2 receptor-induced vasodilation in stroke-prone spontaneously hypertensive rats involves nitric oxide and cGMP-dependent protein kinase. J Hypertens. 2006;24(12):2417-22. doi: 10.1097/01.hjh.0000251902.85675.7e.

10.	Cibi DM, Bi-Lin KW, Shekeran SG, Sandireddy R, Tee N, Singh A, et al. Prdm16 Deficiency Leads to Age-Dependent Cardiac Hypertrophy, Adverse Remodeling, Mitochondrial Dysfunction, and Heart Failure. Cell Rep. 2020;33(3):108288. doi: 10.1016/j.celrep.2020.108288.
